# Access to general practice appointments and sustainable change: a focused ethnographic case study

**DOI:** 10.3399/BJGP.2025.0140

**Published:** 2025-12-16

**Authors:** Helen Atherton, Abi Eccles, Carol Bryce, Annelieke Driessen, Toto Gronlund, Catherine Pope

**Affiliations:** 1 Primary Care Research Centre, University of Southampton, Southampton, UK; 2 Nuffield Department of Primary Care Health Sciences, University of Oxford, Oxford, UK; 3 Warwick Medical School, University of Warwick, Coventry, UK; 4 University of Amsterdam, Amsterdam, the Netherlands; 5 Nuffield Department of Primary Care Health Sciences, University of Oxford, Oxford, UK

**Keywords:** access to primary care, general practice, health services needs and demand

## Abstract

**Background:**

Access to an appointment with a GP is important to patients, but hard to achieve in modern general practice, with general practice delivering more consultations than ever before. Research has focused on discrete systems for managing access in general practice, for example, telephone prioritisation, and these have been demonstrated to be variably successful in managing demand.

**Aim:**

To examine the sustainability of previous attempts to improve access to GP appointments to understand if access systems previously deployed have been adapted, abandoned, or sustained.

**Design and setting:**

This was a focused ethnographic comparative case study in eight English general practices.

**Method:**

Qualitative observation, semi-structured interviews, and documentary analysis were undertaken. The study included 74 patient and 70 staff interviews.

**Results:**

Approaches to managing access are heavily focused on management of demand, and general practices constantly change access systems to try to achieve this. In all the case-study practices, access solutions previously deployed were adapted, rather than abandoned or adopted, usually via ongoing changes or ‘persistent tinkering’. The complexity introduced by these adaptations can be confusing for patients and fuels dissatisfaction, stress, and hostility. Persistent change to access systems creates unintended consequences and significant work for all involved.

**Conclusion:**

Persistent tinkering is a necessary and reasonable response to the challenges of access in general practice. In part this is because the problem is framed as one of managing demand. An alternative approach might investigate what patients want or need and consider how best this could be delivered.

## How this fits in

The long-term sustainability of different systems for managing access in general practice is not understood and this study explored whether previous attempts to improve access to GP appointments have been adopted, adapted, or abandoned in English general practice. There is not a single, sustainable one-size-fits-all access system and persistent tinkering is a necessary and reasonable response to the challenges of access in general practice. In part this is because the problem is framed as one of managing demand rather than providing access. An alternative approach would be to support general practice in applying flexibility to their management of access rather than using top-down approaches to standardise access, and to support patients to help them better navigate the layers of access they encounter.

## Introduction

Access to general practice is a contentious and enduring concern for politicians, policymakers, practitioners, and the public. News media are replete with negative stories about waiting times for GP appointments^1^ and satisfaction rates are falling.^2^ Delays at this front door to the NHS mean late or missed diagnosis and treatment, and provoke inappropriate use of other health and care services.^3^ The national and international policy and research literature abounds with examples of ‘solutions’ to this access crisis, yet the problem persists.^4–6^

Use of general practice varies across Europe^7^ but the UK has seen a significant increase in numbers of consultations^8^ despite a continued decrease in the number of full-time equivalent GPs.^9^ Consultation rates are highest in areas of deprivation,^8,10^ where the GP to patient ratio is lowest, and this exacerbates health inequalities. Added to this, patient presentations have become more complex as the population ages and multimorbidity increases. It is therefore unsurprising that GPs report being stressed and overworked,^11^ and that there is a global crisis in GP recruitment and retention.^12^

Many solutions to the access problem have been tried, including varying appointment availability and length, limiting the number of problems seen per consultation, using telephone^^13,14^^ or online triage,^^15,16^^ diverting patients to other care providers including non-medical staff,^17,18^ and/or offering remote consultation modalities.^19,20^ Many of these approaches have been subjected to formal research evaluation, notably Advanced Access (prioritising same-day appointments),^21^ telephone^13,14^ and online triage,^22–24^ but to our knowledge no one has explored whether previous attempts to improve access to GP appointments have been sustained and adopted, adapted, or abandoned in English general practice. This paper seeks to fill this evidence gap.

## Method

We report this study using the Standards for Reporting Qualitative Research developed by O’Brien *et al*.^^25^^

### Study design and participants

We conducted a team-based focused ethnography, incorporating qualitative observational fieldwork (non-participant observation, informal conversations, and document collection) and semi-structured interviews with staff, patients, and carers at eight English general practices.^26^

Focused ethnography allows for shorter and targeted periods of ethnography, focused on a predetermined topic, in this case access. Although focused, it is also in-depth, allowing for exploration of a given topic using the interpretivist paradigm, and making efficient use of team capacity.^27^

### Sampling of general practices

We sought to recruit eight general practices, in line with previous focused ethnographic case studies examining service delivery in general practice.^28,29^ Across the practices we sought varied levels of deprivation, rural/urban locations, and different numbers of GPs and list size.

To examine whether previous attempts to improve access to GP appointments have been sustained and adopted, adapted, or abandoned, we were informed by a scoping review of previous evaluations of general practice systems, defined as those designed to provide patient access to an appointment for a consultation.^5^

Based on the access system types identified in the review we identified practices that had taken part in research with the following approaches:^5^

- Advanced Access;
- forms of telephone triage (GP/nurse led, telephone first); and
- online consultation.

We were able to recruit six practices that had previously been the subject of formal research.

Two additional practices were sampled to augment geographical coverage and inclusion of practices within the lower deprivation deciles, both being in the 20% to 30% most deprived areas in England. Although these two practices had not been formally involved in previous evaluations, they had tried different access modalities during the COVID-19 pandemic and one was making greater use of Additional Roles Reimbursement Scheme staff,^30^ that is, additional non-GP care providers.

Practices were approached with the assistance of local National Institute for Health and Care Research (NIHR) Clinical Research Networks.

### Procedures for observation fieldwork

The research team collected data from November 2022 to February 2024, spending between 4 and 8 weeks in each practice with duration dependent on practice size. Four researchers collected data from two practices each during the study period. To support the team-based approach, a one-page case-study guide was used as a prompt for researchers that outlined the study requirements and any prompts that may be needed to obtain information from practice staff.

Posters were displayed to inform people when observations were being conducted. Staff, patients, and those accompanying them were able to decline being observed by alerting staff or the researcher.

Observational data collection included periods of familiarisation and targeted observations (such as, focus on reception desk areas) but did not take place in consultation rooms. Researchers had informal conversations with staff when appropriate, observed relevant practice meetings, and collected pertinent documentation, such as, practice policies in electronic form. Fieldnotes were handwritten, contemporaneously or as soon as possible afterwards. To ensure that data were collated in a standardised way, a ‘structured practice summary’ was produced for each practice. It collated information about each practice, included summarised fieldnotes, details about interviewees, and notes about any relevant documentation collected. These were living documents that could be updated throughout data collection and analysis.

### Sampling and procedure for interviews

We sought to interview six to eight staff members per practice, to ensure that all relevant staff members were included in our sample. General practice staff were sampled to represent different clinical and non-clinical roles, and invited to participate via an email from the practice manager or lead GP.

We sought to interview eight to 10 patients per practice, aiming for maximum variation in age, ethnicity, gender, and presence/absence of long-term conditions or disabilities. We used an adaptive sampling strategy, being responsive to the challenges facing each practice. This meant, for example, in a practice that transpired to have a high level of patients not attending booked appointments, we sought to interview patients who had missed an appointment.

Practice staff (usually the practice manager) invited potential patients/carers for interview. Invites were made by email, letter, text message, or in person. Those interested in taking part were given a participant information sheet and invited to provide consent.

Interview topic guides were piloted with members of the study’s patient and public involvement (PPI) panel. Staff interviews explored experiences and decision making surrounding access systems. Patient/carer interviews explored how they made appointments and their experience of changes to access systems. See Supplementary Information S1 for the topic guides. Interviews were scheduled at a mutually convenient time, conducted by telephone, videocall, or in person at the request of the interviewee. Interpreting support was available. Informed consent was obtained from all interview participants. Interviews were digitally recorded, transcribed verbatim by university-approved transcribers, and subsequently de-identified.

### Researcher team and reflexivity

There were three ethnographers on the team, two with a background in sociology and one with a background in anthropology. A senior team member, a medical sociologist, took part in fieldwork alongside the ethnographers. Another senior team member, with a background in anthropology and health services research, led the analysis of the data. The PPI contributor was experienced at public involvement and contributed to analysis throughout. All team members had experience of being involved in healthcare research.

### Data analysis

Data analysis began during data collection and continued until September 2024. Researchers made summaries of key data for each practice as data was collected and the study team read transcripts, fieldnotes, and summary documents, and discussed these to devise a coding frame that iteratively developed as data collection progressed. Multiple team members coded elements of the data to ensure completeness. Further team meetings supported the identification of descriptive themes and explanations, and allowed us to challenge interpretations to ensure the credibility of findings.

Analysis was supported by the preparation of charts outlining key findings^31^ and the ‘one sheet of paper’ analytical approach^32^ — a mind-mapping approach that builds an understanding of the dataset by noting all issues that each section of data raise and finding connections between them. This approach was augmented by the use of timelines outlining the adaptations and changes made to access systems in each practice. Throughout the analysis we drew on insights from relevant theories including those concerning the adoption of innovation into healthcare settings and how these embed.^33,34^ This was particularly relevant when examining adaptations and changes and Levesque’s conceptual framework of access to healthcare, which considers both the healthcare system and the a patient’s ability to access care^35^ that is useful for contrasting data from practices and patients. These theories were used to aid data interpretation.

### Patient and public involvement

PPI shaped the design of our study, with our diverse PPI group of seven people actively involved throughout the study, influencing design, conduct, and dissemination. Our patient public co-investigator (the fifth author) was involved as a full team member throughout the study and contributed to study manuscripts.

## Results

The eight participating general practices varied by list size (5000–33 000), location, and Index of Multiple Deprivation score (from 1 to 10, with 1 being the most deprived) (Table 1). Practices A–F had previously been involved in a research study about access. We conducted 70 staff interviews and 74 patient interviews (Tables 2 and 3, respectively).

**Table 1.** Characteristics of the eight case-study sites

Site ID	List size	Location	IMD score	Previously used access system	Access options at the time of the study
A	33 000	South Central, urban	8	Advanced Access	Telephone, reception desk in person, online triage tool
B	13 000	West Midlands, rural	9	Telephone first	Telephone and online triage tool
C	8000	North-West, urban	5	Telephone first	Telephone, reception desk in person, online triage tool
D	5000	West Midlands, urban	6	Telephone triage by GP or nurse	Telephone
E	20 000	South Central, semi-rural	10	Alternative consultation (email, video, phone)	Telephone, reception desk in person, online triage tool
F	9000	North-East, urban	1	Telephone first	Telephone, online booking, walk-in appointments, online triage tool
G	12 000	Yorkshire, urban	3	N/A (sampled for increased range of deprivation deciles)	Telephone on the day and pre-bookable, online messaging/SMS
H	12 000	Yorkshire, urban	3	N/A (sampled for increased range of deprivation deciles and use of additional roles)	Telephone and online triage tool

IMD = Index of Multiple Deprivation. N/A = not applicable.

**Table 2.** Characteristics of staff taking part in interviews across all case-study sites

Characteristic	Participants, *n* (*N* = 70)
**Age group, years**	
18–29	5
30–39	11
40–49	18
50–59	16
60–69	7
Not recorded	13
**Gender**	
Female	54
Male	15
Not recorded	1
**Role**	
Administrator	4
Assistant practice manager	4
GP partner	19
Healthcare assistant	4
Practice manager	8
Practice nurse	5
Receptionist	16
Receptionist and healthcare assistant	2
Receptionist, supervisor	7
Social prescriber	1
**Years at practice**	<1 to 34

**Table 3.** Characteristics of patients/carers taking part in interviews across all case-study sites

Characteristic	Participants, *n* (*N* = 74)
**Age group, years**	
18–29	6
30–39	9
40–49	5
50–59	11
60–69	14
70–79	16
≥80	7
Missing	6
**Gender**	
Female	45
Male	29
**Long-term condition/s**	
Yes	44
No	13
Not recorded	17
**Disabilities**	
Yes	18
No	21
Not recorded	35
**Ethnicity** ^**a**^	
White British	52
White Irish	2
White Other	2
Asian British	1
Asian Indian	2
British Pakistani	1
Asian Other	2
Black African	1
Other British	1
Other Arab	3
Not recorded	7
**Carer status**	
Yes	12
No	50
Not recorded	12

a Ethnicity collected using self-report and presented here using census categories to protect identity of participants.

All eight general practices had changed their access systems over time. The COVID-19 pandemic had increased the use of remote consultations, removed direct online booking of appointments with GPs, and encouraged the use of online triage platforms. Some practices were trialling new access systems or adaptations while we were conducting our study.

We begin by explaining how the particular focus on demand and urgency shapes access to GPs, and drives ‘persistent tinkering’ or adaptation of access systems.

### Demand and being ‘demanding’

Informed (sometimes tacitly) by economic theory, access was understood by staff in the case-study practices as a supply and demand problem, so management of access was demand focused:

*‘We already know there is completely unlimited demand.’* (Staff [S]70, GP partner, male)

Demand in health care is understood as *‘the level of use at which the perceived marginal health benefits of care equal the marginal cost of accessing care’*.^36^ The challenge in contemporary health systems is that patients’ and healthcare professionals’ perspectives of perceived benefits and costs^36^ are highly variable. Demand may be used as a synonym for ‘healthcare need’ but might equally be regarded as unnecessary, avoidable, or supplier distorted.^36^ Not all patient demand was regarded as legitimate by practice staff:

*‘… it’s so incredibly rare that I see someone and I think, “God, I can’t believe you’ve waited two weeks to see me.” What’s much more common is someone coming in to see me and I think how an earth did you get an appointment.’* (S46, GP partner, female)

Practice staff mobilised the concept of ‘urgency’ as justification for satisfying demand in particular ways, but the meaning of this term was unstable:

*‘During my Monday on* [as] *duty doctor, there’s so many tasks coming through, this is the kind of thing where you look* … *and think, OK that’s not urgent for today, but it needs to be sorted …* [but] *it’s impossible. There are so many more things that are more urgent today that I need to sort.’* (S51, GP partner, male)

Patients also understood that ‘urgency’ provided a threshold for their request for access being met:

*‘I might have had to wait a week, ten days at the worst, but normally it’s within a week, you get an appointment within the week, or if it’s something really urgent, then the same day.’* (Patient [P]54, female, age 50–59 years)

Practices operated multiple access systems. These included allowing appointment booking via telephone (or, less often, online), or requiring the completion of an online form or a telephone consultation before an appointment could be made. Patients could be offered an appointment with other (non-GP) healthcare professionals and/or different appointment types (same day, book ahead, follow-up, health check). Changes to access systems were made in response to national policy directives, local contractual obligations, and/or decisions at practice level. As a result patients often experienced multiple layers of access — as in the example below that offers a call back, the online form, and phone back next week options — following a request for a GP appointment:

*R answers a call, ‘Good morning* [practice name ], *R speaking…’* [She follows a standard script.] *‘Can I take your name? Is it an urgent matter that you need to be seen today?’* [listens] *‘The only thing I have today is … one of our urgent care doctors will call you and if they need to see you they will be able to book you in … If you do e-consult* [form] *someone will be able to help you.’* [listens] *‘Oh, you are not good with computers … Oh, you’ve got no computer. OK’.* [brief exchange] *‘OK you can try again, yes in the middle of next week. Yes, try again’.* (Fieldnotes)

Ultimately, patients contacted the practice seeking an appointment with a GP (sometimes a preferred GP). This appeared, to them, as a simple request, but its accomplishment was complex, not least because the access systems kept changing, as we show below.

### Continual change

In our case-study practices the solution to the problem of demand for GP appointments required what Mol *et al* ^37^ refer to as ‘persistent tinkering’. This constant adaptation helped to make systems, or patchworks of systems together, work *‘in a world full of complex ambivalence and shifting tensions’*.^37^ Adaptation manifested as a long list of ‘things we have tried’ as in this quote:

*‘So when I first started it* [demand] *was uncapped, and we had call volumes of seventy, eighty per person* [clinician] *per day. It was phenomenal and not manageable. And then we capped the calls, can’t even remember what at … just before COVID, but still it was all telephone triage, and we brought patients in as we felt needed … we relied quite a lot on the trainees and the nurse practitioner even for that. During COVID the trainees switched to telephone calls, and so did the nurse practitioner. Then the BMA* [British Medical Association] *guidance came out, saying that we really should cap our calls at twenty-five patient contacts a day … we thought, “Well, we can’t do telephone calls and then face to face”, because you’re really capping it at about fifteen really* [because of the duplication] *… So we talked about it and we’ve now got a hybrid system whereby the patient gets to choose whether they want a telephone call or a face to face* [reducing duplication]*. I think it’s working reasonably well, the patients seem to be loving it. They seem to be choosing reasonably sensibly.’* (S14, GP partner, female)

COVID-19 pandemic risk management measures were cited as a reason for significant changes:

*‘… obviously, that’s changed and been tweaked as COVID’s gone on, and as we’ve come out of COVID, they’ve kept certain things … for example, we’re not pre-booking. We book on the day, unless it’s an e-consult.’* (S18, receptionist, female)

*‘I think the only thing with it is, is that where things have changed since the dreaded COVID, it’s a case that you’re never quite sure who the hell you should go and see and about what.’* (P39, female, age unknown)

But more often small adaptations took the form of ‘workarounds’ or local solutions to immediate access problems. Reception staff were responsible for most workarounds, and we observed them modifying the rules for making an appointment at their own discretion, notably for patients seen as especially vulnerable, such as, people with disabilities, those with mental health conditions, or with poor English:

*‘*[the receptionists] *will come and say, “She’s been crying”*, *and I will release* [an appointment slot] *sometimes, if I think it’s justifiable, or if they really are, you know, quite distraught.’* (S57, receptionist, supervisor, female)

These micro-level adaptations often shifted patients between different access systems, for example, when the patient phones in but there are no appointments left they are told to complete an online form. Access becomes what human factors analysts call ‘work as done’ rather than ‘work as imagined’.^38^ Work as imagined was inscribed in national policies about access (dictating, for example, in May 2023 that GPs should provide more face-to-face appointments) but was often in tension with what GPs and other staff felt could be offered:

*‘I think the constraints of the new GP contract may undermine some of the good practice that we’ve developed over the last couple of years even though the intention is to improve access* […] [If] *we don’t have any more appointments we have to then push the patient to go to 111, which doesn’t help them. It probably doesn’t help us, ’cause … the patient’s gonna come back to us the next day having been through 111. But the contract requires us to provide them with an appointment or signpost them to a service immediately as they ring up.’* (S27, GP partner, male)

### Dissatisfaction, stress, and hostility

Persistent tinkering with access systems made it difficult for patients to understand how to get an appointment, especially if they visited the clinic infrequently. Trying to obtain a GP appointment was challenging and getting one felt like a significant achievement:

*‘I know a lot of people who are patients at this surgery and they all feel you can never get an appointment. You are lucky if you can. Today I was lucky. I think it was a cancellation.’* (P56, patient, female, aged 60–69 years)

*‘Today — wonderful: I rang at ... usually you ring at eight o’clock and you redial, and redial, and redial for half an hour, but they’ve changed the system now and they’ve got an answerphone that tells you you’re twenty-sixth in the queue, I was this morning, and I thought, “No, I’m going to hang on.” … And it was fifteen minutes … it went: twenty-six, twenty-five, twenty-three, seventeen, fifteen, then it went to ten, six, and one … that was it.’* (P65, female, age 70–79 years)

When patients do not obtain an appointment, they can become hostile and angry. Staff described verbal and physical abuse directed at reception staff in particular. Reception staff talked about the increasing stress of their role related to these difficult interactions:

*‘The patient phoned up about four times in the afternoon, got quite rude with receptionist, and … in the end, the receptionist said, “I’m really sorry, I might have to terminate this call.” She said she had a string of patients waiting and he was just being really rude and shouting on the phone.’* (S30, practice manager, female)

*‘They’re quite demanding at times. I think that’s got a bit easier now. You know, ’cause when they’d come in and they’d want an appointment, they used to get really quite, some can get really quite irate and rude.’* (S23, practice manager, female)

Interestingly, some GPs were less aware of these access challenges:

*‘We want to make sure patients are accessing the service when they need to. It’s very rare that I hear that a patient couldn’t get through and wasn’t seen, and there was a problem.’* (S45, GP partner, male)

Access challenges fuelled dissatisfaction. Staff noted that:

*One minute people were clapping for them* [to show support for healthcare professionals during the COVID-19 pandemic] *then on the next they were complaining. They feel it is very much driven by the media, and abuse has just got worse.* (Fieldnotes)

Patients felt their expectations were not met:

*‘… my daughter … she has a high temperature … when I call the GP for help, they’re really, not really, helpful, but just ask me to observe and wait and find more information … It* [is] *quite a fall behind my expectation to get the assistance in this way.’* (P30, patient, female, aged 50–59 years)

Continual change to access systems meant that patients and general practice staff perspectives were not aligned, leading to dissatisfaction, stress, and sometimes hostility. There were other consequences associated with persistent tinkering, not always desired or intended, as we show next.

### Unintended consequences

Managing access produced a number of unintended consequences, in addition to the dissatisfaction and hostility noted above. Unintended consequences are positive or negative effects that were not planned or envisioned^22^ and they have been previously described in relation to the implementation of online consultation systems and allowing patient access to their medical record.^22,39^

One positively viewed consequence of the COVID-19 pandemic was to allow changes that were previously seen as unpalatable to patients to be introduced, notably online triage and consultation systems:

*‘We made the conscious decision that we were gonna go digital first. COVID provided a very good reason to, to suddenly do that … because of COVID cancelling all the face-to-face appointments, we could clear the books and actually start on a day-to-day turnover. Which we’ve maintained. We’re generally booking things on the day only, if someone rings up on the day now … which is good for on-the-day access,* [but] *not necessarily so good for patients who want to book two, three weeks ahead.’* (S27, GP partner, male)

However, these new access systems were not without problems. Online triage and online consultation tools, originally designed to provide 24/7 access to leave a message,^16^ were turned off outside of office hours. Reasons given included not only patient safety concerns, but also the need to manage demand:

*‘We used to have them* [patient-facing online triage form] *on twenty-four hours, but we had to change that, because it was just unmanageable. We couldn’t keep up with the demand, and we didn’t have enough appointments to go around either. So, now … they’re just* [open in] *core working hours … they come on at eight-fifteen and they’re switched off at half-six. They’re not on over the weekend, and we switch them off* [on] *bank holidays and Christmas and Easter.’* (S26, practice manager, female)

All the practices we studied had moved through several iterations of different access approaches. All were striving to manage demand, but none appeared to have succeeded completely. Adaptation was the norm as one GP explained:

*‘Adaptation is almost inevitable. We’re not using a purely digital first approach which is how we intended it to be. I suppose any system that you design never, never fits … exactly so you adapt as you go.’* (S27, GP partner, male*)*

To a certain extent, patients were also adapting their approaches as they obtained new information on how they could best achieve access:

*‘And I’ve only recently, and I mean very recently, within the last couple of months, found that when you’re in that* [telephone] *queue you can, you can type in a code number and they will either phone you back, or you can turn up at the surgery at eight a.m. They open the shutters and you can ask to see a doctor.’* (P49, female, age 60–69 years)

Each adaptation could create new problems. For example, practices had discovered that they needed to manage the availability of future GP appointments carefully, as booking appointments too far ahead increased the number of patients who did not attend (DNA). They adapted their access systems accordingly:

*‘We have a number of* [appointments] *locked for on-the-day demand and then the rest can be used … or booked whenever. We have some which are pre-bookable and that’s normally sort of three to five days, the reason being that we felt any longer than that and we ended up with higher DNA rates.’* (S54, GP partner, male)

But an unintended consequence of restricting future booking was to drive up demand for urgent appointments (and some patients described exaggerating symptoms in order to get an appointment), fuelling the sense of insatiable demand.

## Discussion

### Summary

We have shown that approaches to managing GP access are heavily focused on management of demand. None of the practices we studied had fully adopted a single-access system, but nor had they completely abandoned systems tried previously. Our case-study practices constantly adapted their access systems to try to meet demand. The resulting layering and complexity of access was confusing for patients, and created dissatisfaction, stress, and hostility, and often had other unintended consequences. Some adaptations to access systems are driven by externalities such as COVID-19 or policy directives, but many are derived from local, everyday attempts to manage the access problem. Persistent tinkering with access systems creates considerable work for all involved, staff implementing changes and patients navigating these, but it seems that there is not a single, sustainable one-size-fits-all access system.

### Strengths and limitations

We conducted a comparative case study in eight English practices, and sought to represent areas of high deprivation, and rural and urban areas, as well as different practice sizes. The detail derived from this focused ethnography offers transferable findings, but not the predictive probability offered by other study designs. We can say that other practice access systems will exhibit adaptation and the kinds of challenges we have identified, but there will likely be local variants in the form that persistent tinkering takes, and the consequences that ensue.

The six practices that had taken part in a previous evaluation of access had introduced changes at different time points (the earliest being Advanced Access in 2003). In some cases, there was little institutional memory of all the changes in access approaches since the original study, but we believe we have captured the core adaptations tried. Our fieldwork was conducted in the aftermath of the COVID-19 pandemic, which had an impact on access systems in all eight practices but, as we have shown, in varying ways. None of the access systems in place were completely duplicated in other practices, confirming our finding that adaptations are locally differentiated.

### Comparison with existing literature

A recent review of access systems in general practice used since 1984^6^ identified that numerous diverse approaches to access have been tried including those examined in this study, and these have led to unintended consequences, an example being online consultation that can improve access for some but causes deterioration of access for others. This historical view consolidates our finding that change has been ever present, and that consequences of different systems are not always anticipated or desirable, affecting different patients very differently.

A recent UK-based study of access to general practice devised a conceptual framework outlining access as the ‘human fit’ between the needs and abilities of patients (to perceive, seek, reach, afford, and engage with the care needed), and the abilities and capacity of general practice staff (to deliver approachable, acceptable, accommodating, affordable, appropriate services).^40^ Our findings similarly demonstrate that ‘human fit’ is currently missing in general practice, and we concur that this concept should inform attempts to improve access.

The persistent tinkering^37^ with access systems might be said to resemble the construct of ‘reflexive monitoring’ captured in normalisation process theory (NPT),^33^ although reflexive monitoring implies an intention to review and improve on a practice and the adaptation we observed was not deliberate or formalised. NPT and its sister theory, the non-adoption, abandonment, scale-up, spread, and sustainability framework,^34^ focus attention on the work required to implement change. However, there is a lack of formality in the change we observed in general practice that implies that these frameworks may not be appropriate for capturing what is happening in access to general practice.

### Implications for practice

There is a clear argument for allowing general practices the resources, time, and space to adapt access arrangements for their populations. Single-access solutions tried in previous research and favoured by politicians and high-level policymakers have not ‘stuck’ or solved the access problem. Persistent tinkering is a necessary and reasonable response that should be supported.

There is a clear need for the management of expectations of staff and patients. Patients and the public need to understand access systems, supported where necessary to navigate these or work around them. General practices may need to challenge their thinking about patient needs and wants in relation to access and consider how they can humanise a process that has become complex and technical. Policymakers could play their part here too, understanding that when patients seek to make an appointment with their GP they may struggle with the expectation that they can use digital tools or will be happy to see a care provider who is not their GP.

There is a question about whether access to GP appointments should be focused so exclusively on the management of demand. This focus has delivered systems that require constant adaptation, provoke dissatisfaction, and has encouraged practices and policymakers to continually seek new initiatives that will improve access. Without better resourcing (notably to address the shortfall in numbers of GPs) practices will be obliged to continue expending effort and resources on tinkering or adapting access systems. An alternative approach would be to support general practice in applying flexibility to their management of access rather than using top-down approaches to standardise access, and to support patients to help them better navigate the layers of access they encounter.

## Data

All data requests should be submitted to the corresponding author for consideration. Access to all de-identified data may be granted following review.
